# Outcomes of two-stage type II hybrid aortic arch repair in elderly patients with acute type A aortic dissection

**DOI:** 10.1038/s41598-024-51784-3

**Published:** 2024-01-17

**Authors:** Jun Xiang, Ling He, Tailuan Pen, Donglin Li, Shuliang Wei

**Affiliations:** 1 Department of Cardiovascular Surgery, Affiliated Hospital of North Sichuan Medical College, Nanchong, 637000 Sichuan China; 2 Department of Pediatrics, Affiliated Hospital of North Sichuan Medical College, Nanchong, 637000 Sichuan China

**Keywords:** Cardiology, Diseases, Health care

## Abstract

Acute type A aortic dissection (a-TAAD) is a severe disease characterized by high mortality, which can be fatal in elderly patients. The objective of this study was to investigate the safety and efficacy of two-stage type II hybrid aortic arch repair (HAR) in elderly patients with acute type A aortic dissection (a-TAAD). This was a single-center, retrospective study involving 119 patients with a-TAAD, including 82 males and 37 females, aged 22–81 years old. Eighty-eight patients underwent total aortic arch replacement (TAR) with frozen elephant trunk (FET) implantation (TAR with FET group) and 31 patients underwent two-stage type II HAR (HAR group). Propensity score matching was applied to adjust for preoperative data, and match 25 pairs. The preoperative, perioperative, postoperative and follow-up data were recorded. Fifteen patients died during the perioperative period; 13 cases were in the TAR with FET group and 2 cases were in the HAR group. The age, body mass index, cerebral infarction, renal insufficiency were significantly higher, and the 24-h fluid drainage, the incidence of acute liver injury, acute kidney injury and pulmonary infection were lower in the HAR group (all *P* < 0.05). Moreover, the mechanical ventilation time, intensive care unit time, hospital stay time were shorter in the HAR group (all *P* < 0.05). The follow-up period ranged from 12 to 54 months, with 7 deaths (9.3%) in the TAR with FET group and 2 deaths (6.9%) in the HAR group. The true lumen of the aortic arch and the middle descending thoracic aorta were larger and the false lumen thrombosis rates of the middle descending thoracic aorta and renal artery level were higher in the HAR group (all *P* < 0.05). Two-stage type II HAR is a safe and effective method for the treatment of elderly patients with a-TAAD. It may be a good choice for elderly patients with a-TAAD and comorbidities.

## Introduction

Acute type A aortic dissection (a-TAAD) is a serious type of acute aortic syndrome. It is one of the most fatal diseases with an acute onset and high mortality. Delayed surgical treatment may increase the mortality rate by 1–2% per hour within 48 h, which may reach approximately 90% within 1 week^1,2^. As the aging population increases, the morbidity of a-TAAD among elderly people has been increasing. Considering that a-TAAD responds poorly to medical therapy, emergency surgery remains the first-line treatment option^3^. Conventional total aortic arch replacement with frozen elephant trunk (TAR with FET) implantation, also called the Sun’s procedure, has been widely considered the best surgical approach for a-TAAD^4^. However, this procedure is complicated and requires hypothermic circulatory arrest (HCA), which increases the risk of postoperative complications and mortality^5^. The mortality rate is much higher in elderly patients or patients with more preoperative complications^6^. With the rapid development of interventional techniques in recent years, new concepts in the surgical treatment of a-TAAD have been proposed. Hybrid surgery has become a new option for the treatment of a-TAAD^7^. Some studies have reported that type II hybrid aortic arch repair (HAR) achieved good short- and medium-term effectiveness in the treatment of a-TAAD^8–10^. However, they all use one-stage HAR, which is performed in hybrid operating rooms; however, most medical facilities in developing countries lack hybrid operating rooms. Two-stage HAR may be an alternative approach for elderly a-TAAD patients or patients who cannot tolerate HCA. Currently, few studies have explored the safety and efficacy of two-stage HAR. In this study, we compared short- and medium-term effects of two-stage HAR and conventional TAR with FET in the treatment of a-TAAD to explore the safety and efficacy of two-stage HAR in the treatment of elderly patients with a-TAAD and provide a theoretical basis for the treatment of elderly patients with a-TAAD at the grassroots level.

## Materials and methods

### Patients

**Figure 1:**
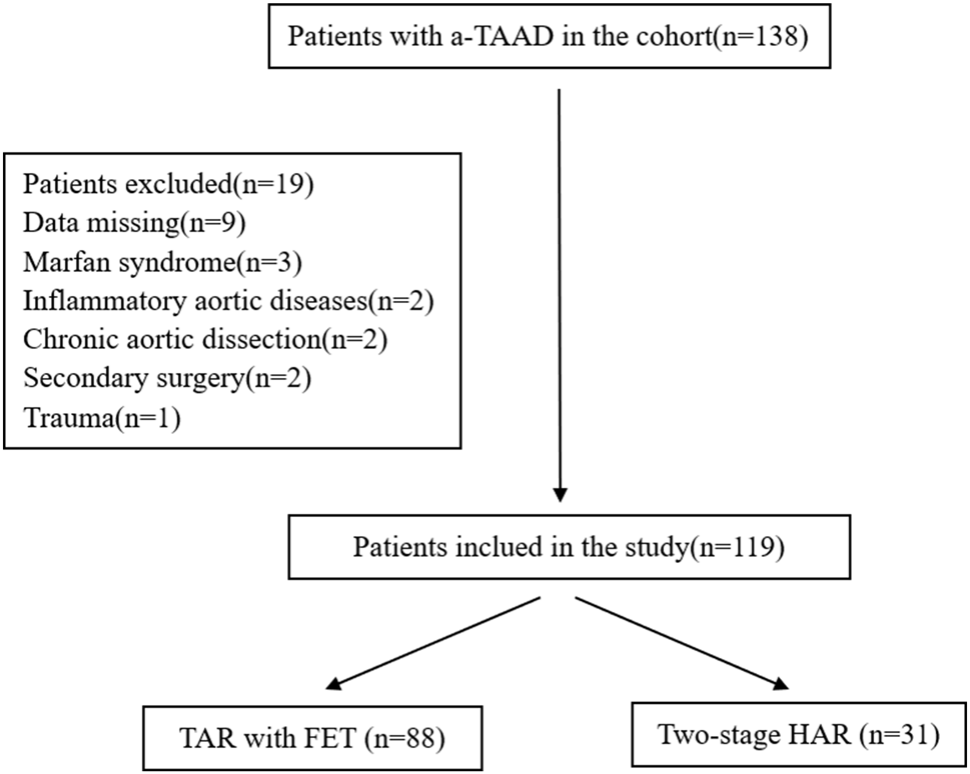
Flow chart for patients enrollment and study design. a-TAAD: Acute type A aortic dissection; TAR: total aortic arch replacement; FET: frozen elephant trunk.

Patients with a-TAAD who underwent two-stage HAR surgery or TAR with FET surgery at the Affiliated Hospital of North Sichuan Medical College from January 2018 to October 2022 were retrospectively analyzed. All patients were diagnosed as a-TAAD by total thoracic and abdominal aorta computed tomography angiography (CTA), and surgical indications were recommended according to the relevant guidelines^3^. Patients with inflammatory aortic diseases, Marfan syndrome, connective tissue disease, chronic aortic dissection, secondary surgery, pregnancy, trauma and significant missing medical records were excluded. A total of 138 cases of AAD were enrolled in this study. After applying the exclusion criteria, 119 patients with AAD were enrolled, 88 cases of TAR with FET and 31 cases of two-stage HAR (Fig. 1). TAR with FET surgery was recommended for patients aged < 60 years old, while two-stage HAR surgery was recommended for patients aged ≥ 60 years old, who had preoperative cerebral infarction, malperfusion syndrome, severe chronic obstructive pulmonary disease and renal insufficiency. However, the specific surgical procedure was determined through consultation between family members and the surgeon. This study was approved by the Medical Ethics Committee of the Affiliated Hospital of North Sichuan Medical College (No. 2021 ERA098). All participants provided written informed consent to participate after receiving a plenary explanation of the study.

### Surgical procedure

#### TAR with FET surgery

**Figure 2:**
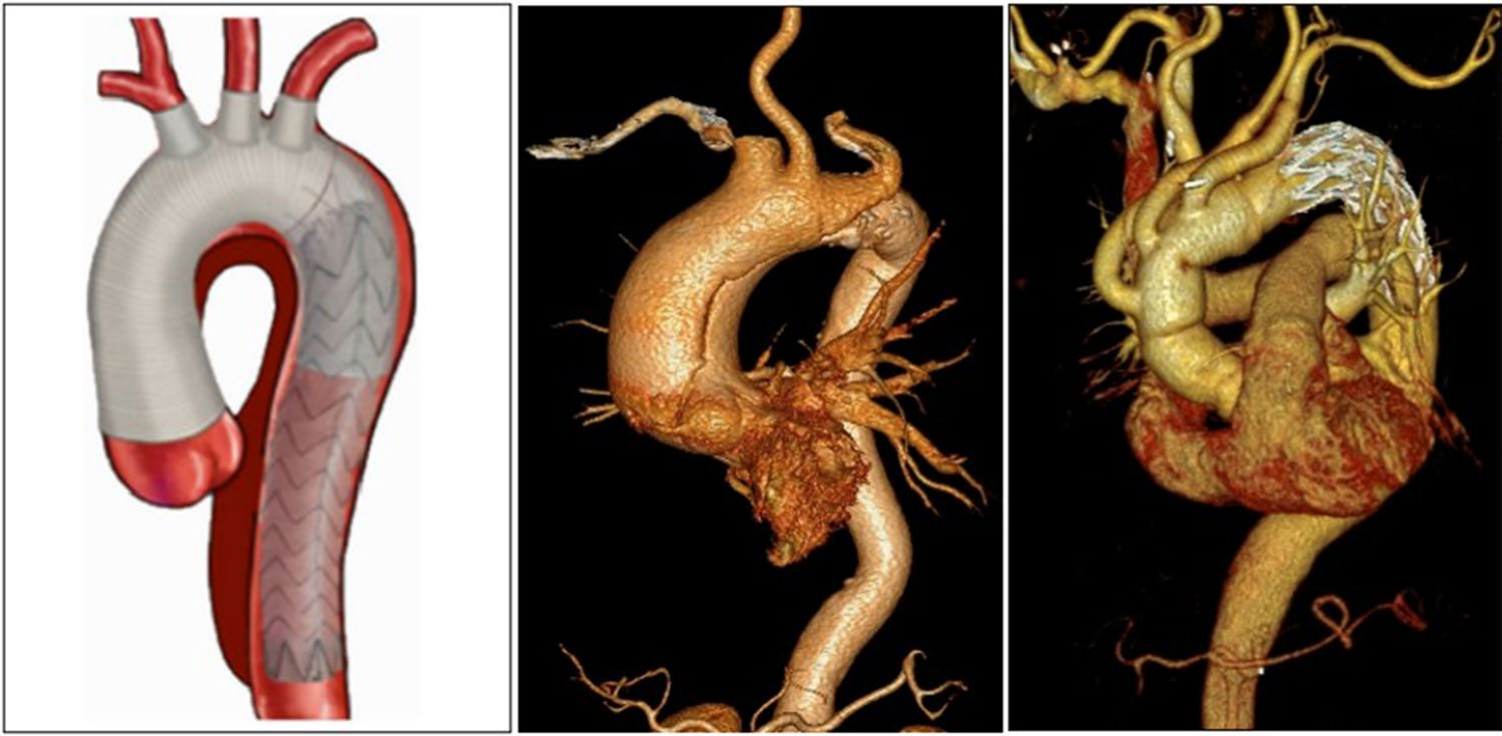
TAR with FET surgery procedure. TAR: total aortic arch replacement; FET: frozen elephant trunk.

The surgery was performed through a median sternotomy under cardiopulmonary bypass (CPB) with general anesthesia. CPB was established through the femoral artery, right axillary artery or brachiocephalic trunk artery and right atrium cannulation, and the ascending aorta was clamped when the nasopharyngeal temperature was 32°. The ascending aorta was dissected longitudinally to remove the false lumen thrombus, and the left and right coronary arteries were perfused with myocardial protective solution. Ascending aorta replacement, Bentall, Wheat, David, and sinus reconstruction with ascending aorta replacement were conducted according to the aortic root lesions respectively. When the nasopharyngeal temperature dropped to 20–24 °C, and the anus temperature dropped to 25 °C, circulation was stopped, and anterograde cerebral perfusion (5–10 ml/kg/min) from the right axillary artery or brachiocephalic trunk artery was performed. The aortic arch was severed between the left subclavian artery and the left common carotid artery to remove the thrombus. The stent elephant trunk was implanted in the descending aorta through the true lumen. The distal end of the four-branch artificial blood vessel was anastomosed with the proximal end of the stent elephant trunk and the descending aortic wall continuously through slide wire. Perfusion was restored to the lower body through the four-branch of the artificial blood vessel. Next, the brachiocephalic trunk artery and the left common carotid artery were anastomosed with the branches of the artificial blood vessel, respectively. The proximal end of the artificial blood vessel was anastomosed to the ascending aorta. The occluding clamp was released after venting. The left subclavian artery was anastomosed after heartbeat recovery. After the circulation was stabilized, the CPB machine was withdrawn, hemostasis was fully stopped and the incision was closed (Fig. 2).

#### Two-stage HAR surgery

**Figure 3:**
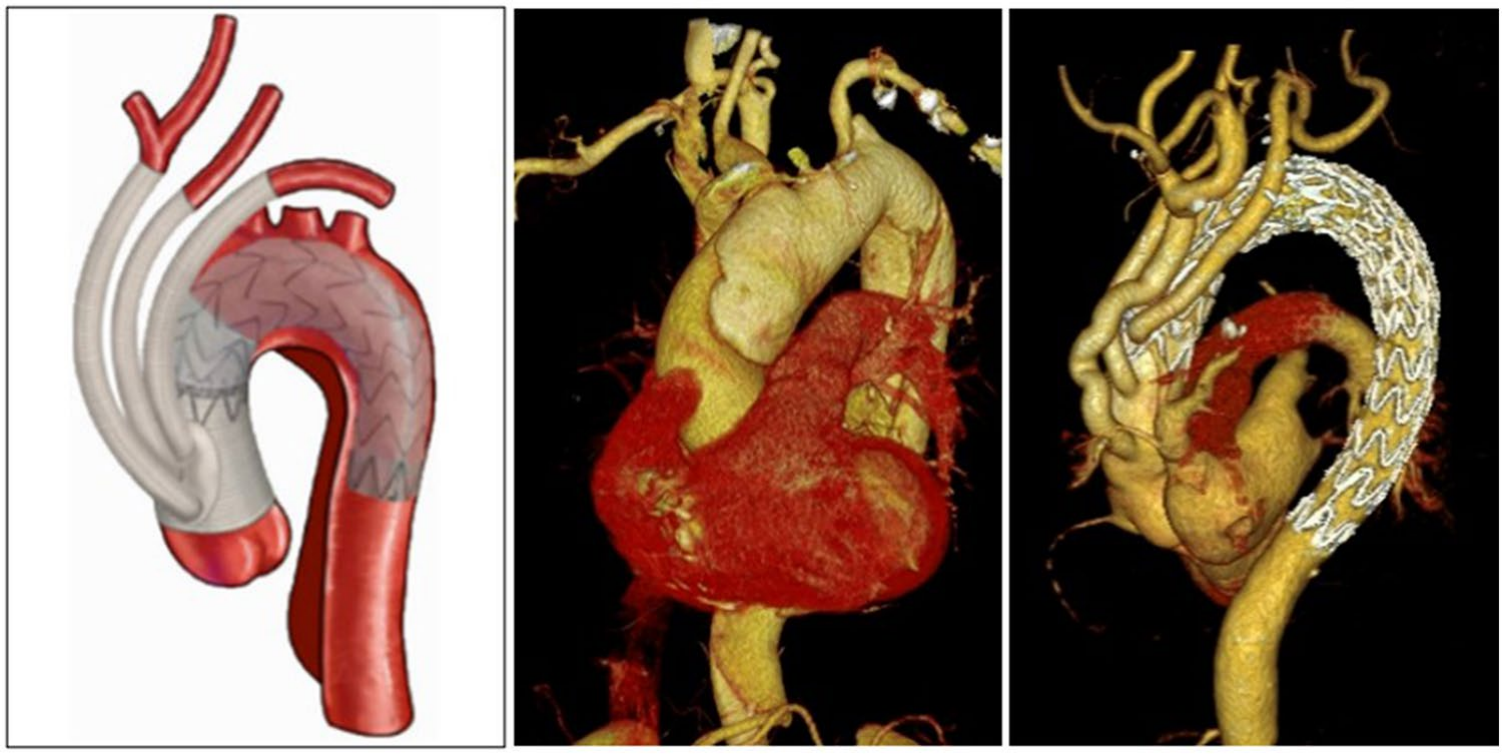
HAR surgery procedure. HAR, hybrid aortic arch repair.

The surgery was also performed through a median sternotomy under CPB under general anesthesia. The CPB was conducted using a similar approach to that of TAR with FET surgery. An occluding clamp was applied to block the aortic arch between the brachiocephalic trunk artery and the left common carotid artery when the nasopharyngeal temperature was 32°, the brachiocephalic trunk artery was clamped too. The ascending aorta was dissected longitudinally to remove the false lumen thrombus, and the left and right coronary arteries were perfused with myocardial protective solution. The surgical methods for aortic root were similar to those of TAR with FET surgery. The distal ascending aorta was severed near the brachiocephalic trunk artery. The anastomotic area of the proximal aortic arch was sandwiched with an artificial vascular ring in and a felt strip out, and the distal end of the four branches of the artificial blood vessel was anastomosed with the sandwich, the distance between the branch vessels and anastomosis should be more than 15 mm. The proximal end of the artificial blood vessel was anastomosed to the ascending aorta. The occluding clamp was released after venting to restore blood flow to the heart. Subsequently, the left subclavian artery, left common carotid artery and brachiocephalic trunk artery were anastomosed with the branches of the artificial blood vessel, respectively. If the patient had a short ascending aorta, the fourth branch of the artificial blood vessel was anastomosed to the left subclavian artery. Distal anastomosis and distal branch artificial vessels were labeled with titanium clips. Once circulation was stabilized, the CPB machine was withdrawn, hemostasis was fully stopped and the incision was closed. After surgery, patients were transferred to the intensive care unit (ICU) and given relevant treatment, and thoracic endovascular aortic repair (TEVAR) was performed in an interventional radiotherapy room once hemodynamic stability was achieved. TEVAR was performed if two of the following three surgical indications were met: (1) hemodynamic stability, systolic blood pressure > 90 mmHg or mean arterial pressure > 60 mmHg; (2) recovery of consciousness, no cerebral complications; (3) drainage flow < 50 ml/h. TEVAR was performed through the original femoral artery cannulation incision under general anesthesia in the intervention radiotherapy room. Aortography was performed first to check the artificial blood vessel, anastomosis, the anchoring area and dissection rupture. The stent was selected based on the diameter of the anchoring area measured through digital subtraction angiography and the diameter of the artificial blood vessel during the operation, and stent magnification was 10–15%. A restricted stent was placed in the appropriate position of the descending aorta and then the main stent was placed in the aortic arch, with the proximal end of the stent in the artificial blood vessel. When the stent was completely released, angiography was performed to confirm the position, shape and occlusion of the aortic dissection rupture. After the surgery, the patients were transferred to ICU for further treatment (Fig. 3).

#### Postoperative treatment

Patients were provided with ventilator support for respiratory assistance, vasoactive medications to ensure stable blood pressure and heart function, and broad-spectrum antibiotics for infection prevention. Upon achieving sustained stability in hemodynamic parameters without serious complications, they were safely transitioned to the general ward for continued recovery (such as pulmonary infection, respiratory failure, renal failure, cardiac failure, disturbance of consciousness, among others). Intra-aortic balloon pump (IABP) and continuous renal replacement therapy (CRRT) were selected based on patient complications. Patients with biological valve replacement received warfarin for 6 months while those with mechanical valve replacement received the same drug for a lifetime, and the international normalized ratio (INR) was measured regularly and maintained between 1.8 and 2.0. Patients were recommended to undergo an aortic CTA examination before discharge.

#### Data collection and follow‑up

Before data collection, a unified and applicable clinical statistical scale was designed. Preoperative-related variables (including demographic data, laboratory tests, cardiac ultrasound results and CTA results), intraoperative data (including root treatment, operation time, CPB time, aortic cross-clamping (ACC) time, HCA time, transfusion, etc.), perioperative major complications (including 24-h drainage, thoracotomy hemostasis, cardiac dysfunction, acute renal injury, acute liver injury, pulmonary infection, brain dysfunction, ventilator support time, ICU stay time, hospital stay time, etc.) and in-hospital mortality were routinely recorded.

Follow-up data such as survival status, death time, cause of death, reoperation, internal leakage and radiographic outcomes were obtained from outpatient visits combined with telephone calls. Aortic CTA was performed 3 months after the operation and annually thereafter to evaluate the aortic remodeling, including the change in the aortic diameter and false lumen thrombosis extent. The aortic diameter was measured at the ascending aorta level, aortic arch level, middle descending thoracic aorta level, renal artery level and middle abdominal aorta level. False lumen thrombosis was evaluated at the distal arch level, middle descending thoracic aorta level, renal artery level and middle abdominal aorta level. False lumen thrombosis was defined as a thrombosis rate of > 80% or complete obliteration of the false lumen. Reoperation refers to the distal interventional treatment due to the small true lumen or the continuous enlargement of the false lumen caused by distal residual aortic dissection. The definition of Low-cardiac-output syndrome (LCOS) includes decreases in the cardiac index (CI) to < 2.0 L/min/m2 and a systolic blood pressure of < 90 mmHg, in conjunction with signs of tissue hypoperfusion (cold periphery, clammy skin, confusion, oliguria, elevated lactate level) in the absence of hypovolemia. The definition of acute kidney injury (AKI) includes: (1) gradually decreased urine output < 0.5 mL/kg/hour, for consecutively three hours with a poor response to diuretics; and (2) plasma creatinine > 110 µmol/L or exceeding the baseline for over > 50%. The definition of transient neurological dysfunction (TND) include conscious disturbances (including coma, lethargy, paralysis, etc.), sensory or motor impairment, and complete disappearance of all neurological damage symptoms before discharge. And the definition of permanent neurological dysfunction (PND) include new onset of coma, sensory or motor impairment, and any neurological damage symptoms that did not completely disappear before discharge.

### Statistical analysis

SPSS 22.0 was used for statistical analysis. Continuous variables were expressed as $\overline{x}$ ± *s*, and Student’s *t *test or Wilcoxon rank-sum test was used for comparisons between groups. Dichotomous variables were expressed as frequency (rate or percentage), and the chi-square test or Fisher’s exact test was performed to compare groups. Propensity score matching (PSM) was applied to adjust for preoperative data, and 25 pairs were matched successfully. Relevant variables before and after PSM were compared. Survival data and reoperation rate were analyzed using Kaplan–Meier survival curve analysis and the log-rank test. A two-tailed *P* value < 0.05 was considered statistically significant.

### Ethics statement

The studies involving human participants were reviewed and approved by Medical Ethics Committee of the Affiliated Hospital of North Sichuan Medical College ((No. 2021 ERA098)). All the methods were carried out in accordance with relevant guidelines and regulations. And all participants provided written informed consent after receiving a plenary explanation of the study.

## Result

### Baseline characteristics

Before PSM, age (63.1 ± 5.9 vs. 56.3 ± 10.7 years, *P* = 0.001), body mass index (25.1 ± 1.9 vs. 24.2 ± 1.7 kg/m^2^, *P* = 0.016), cerebral infarction (19.3 vs. 3.6%, *P* = 0.004) and renal insufficiency (16.1 vs. 2.3%, *P* = 0.015) were significantly higher in the HAR group than in the TAR with FET group. There were significant differences in sex, hypertension, coronary artery disease, diabetes, chronic obstructive pulmonary disease and indexes of laboratory markers and echocardiography between the two groups (all *P* > 0.05). After PSM, the preoperative data were well-balanced (Table 1).

**Table 1:** Demographic and preoperative data of the two groups.

	Before PSM (n = 119)				After PSM (n = 50)			
	TAR with FET (n = 88)	HAR (n = 31)	t/χ^2^	*P* value	TAR with FET (n = 25)	HAR (n = 25)	t/χ^2^	*P* value
Gender, male (n, %)	59 (67.1)	23 (74.2)	0.547	0.460	17 (68.0)	18 (72.0)	0.095	0.758
Age (years)	56.3 ± 10.7	63.1 ± 5.9	− 3.357	0.001	60.7 ± 8.9	62.4 ± 5.5	− 0.812	0.421
BMI (kg/m^2^)	24.2 ± 1.7	25.1 ± 1.9	− 2.457	0.016	24.4 ± 1.7	24.9 ± 1.7	− 1.029	0.309
WBC (× 109/L)	12.4 ± 3.5	12.1 ± 3.6	0.407	0.684	12.6 ± 3.5	12.3 ± 3.3	0.312	0.757
RBC (× 1012/L)	4.7 ± 1.1	4.5 ± 1.0	0.891	0.375	4.6 ± 1.1	4.5 ± 1.1	0.321	0.749
PLT (× 109/L)	175.6 ± 38.9	168.8 ± 36.1	0.852	0.396	174.3 ± 37.7	169.6 ± 37.1	0.444	0.659
Hb (g/L)	124.3 ± 19.4	121.4 ± 18.8	0.721	0.472	121.8 ± 18.9	122.1 ± 18.1	− 0.057	0.955
D-dimer (mg/L)	10.4 ± 2.5	11.2 ± 2.4	− 1.548	0.124	10.8 ± 2.3	10.9 ± 2.1	− 0.161	0.873
CRP (mg/L)	11.1 ± 3.5	11.8 ± 3.3	− 0.971	0.333	11.2 ± 2.8	11.7 ± 3.0	− 0.609	0.545
ALT (U/L)	37.3 ± 17.4	38.9 ± 18.5	− 0.433	0.665	39.4 ± 14.3	38.5 ± 16.7	0.205	0.839
AST (U/L)	40.3 ± 18.4	37.5 ± 19.1	0.721	0.472	38.2 ± 16.4	37.7 ± 17.8	0.103	0.918
ALB (g/L)	39.4 ± 4.3	37.9 ± 3.8	1.719	0.088	38.7 ± 4.1	38.1 ± 3.8	0.537	0.694
TB (μmol/L)	14.3 ± 3.3	14.6 ± 3.3	− 0.435	0.664	14.2 ± 3.2	14.5 ± 3.3	− 0.326	0.745
Cr (μmol/L)	83.4 ± 37.5	97.2 ± 40.5	− 1.725	0.087	87.5 ± 38.4	96.5 ± 39.7	− 0.814	0.419
cTnT (pg/ml)	8.9 ± 4.1	10.3 ± 5.1	− 1.452	0.149	9.2 ± 4.1	10.1 ± 4.5	− 0.771	0.445
CK-MB (ng/ml)	11.3 ± 4.1	11.7 ± 4.3	− 0.461	0.646	11.4 ± 4.1	11.6 ± 4.2	− 0.170	0.865
NT-proBNP	187.6 ± 110.5	230.7 ± 126.4	− 1.797	0.075	195.5 ± 117.5	218.6 ± 125.5	− 0.672	0.505
Hypertension (n, %)	78 (88.6)	27 (87.1)	0.052	0.891	22 (88.0)	23 (92.0)		0.668*
CHD (n, %)	7 (7.9)	4 (12.9)	0.669	0.413	4 (16.0)	3 (12.0)		1.000*
Diabetes (n, %)	11 (12.5)	5 (16.1)	0.283	0.595	5 (20.0)	4 (16.0)	0.136	0.713
CI (n, %)	3 (3.4)	6 (19.3)	8.338	0.004	2 (8.0)	4 (16.0)		0.667
COPD (n, %)	10 (11.4)	7 (22.6)	2.356	0.125	5 (20.0)	5 (20.0)	0.000	1.000*
RI (n, %)	2 (2.3)	5 (16.1)	5.915	0.015	2 (8.0)	4 (16.0)		0.067*
Smoking (n, %)	45 (51.1)	18 (58.1)	0.442	0.506	14 (56.0)	15 (60.0)	0.082	0.774
Drinking (n, %)	34 (38.6)	14 (45.1)	0.406	0.524	9 (36.0)	10 (40.0)	0.085	0.771
LVEF (%)	59.5 ± 4.5	58.7 ± 4.7	0.507	0.613	59.3 ± 4.4	59.1 ± 4.5	0.158	0.874
IVST (mm)	12.7 ± 1.6	12.8 ± 1.7	− 0.294	0.769	12.9 ± 1.5	12.9 ± 1.7	0.000	1.000
AI (n, %)	35 (39.8)	14 (45.2)	0.275	0.600	9 (36.0)	11 (44.0)	0.334	0.564
Pericardial effusion (n, %)	16 (18.2)	8 (25.8)	0.868	0.363	5 (20.0)	7 (28.0)	0.439	0.508

*PSM* propensity score-matched, *TAR* total aortic arch replacement, *FET* frozen elephant trunk implantation, *HAR* hybrid II aortic arch replacement, *BMI* body mass index, *WBC* white blood cell, *RBC* red blood cell, *PLT* platelets, *Hb* hemoglobin, *CRP* C-reactive protein, *ALT* alanine transaminase, *AST* aspartate aminotransferase, *ALB* albumin, *TB* total bilirubin, *Cr* creatinine, *cTnT* troponin T, *CK-MB* creatine kinase MB, *NT-proBNP* N-terminal pro brain natriuretic peptide, *CHD* coronary artery disease, *CI* cerebral infarction, *COPD* chronic obstructive pulmonary disease, *RI* renal insufficiency, *LVEF* left ventricular ejection fraction, *IVST* interventricular septum thickness, *AI* aortic insufficiency.

*Indicates Fisher's exact test.

### Intraoperative data

Before PSM, the operation time (6.7 ± 0.9 vs. 7.1 ± 1.2 h, *P* = 0.093), CPB time (147.5 ± 27.4 vs. 169.1 ± 33.5 min, *P* < 0.001), ACC time (94.5 ± 15.6 vs. 125.6 ± 19.5 min, *P* < 0.001) and HCA time (0 vs. 12.4 ± 1.9, *P* < 0.001) were significantly shorter in the HAR group than in the TAR with FET group. Besides, erythrocyte (3.1 ± 1.1 vs. 4.4 ± 0.9 U, *P* < 0.001), plasma (440.7 ± 196.1 vs. 585.4 ± 205.6 ml, *P* < 0.001) and platelets (0.41 ± 0.21 vs. 0.64 ± 0.25 U, *P* < 0.001) transfusions were much less during intraoperative in the HAR group than in the TAR with FET group. The statistical results after PSM were consistent with those obtained before PSM (all *P* < 0.05). There were no significant differences in the aortic root surgery procedure between the two groups, both before and after PSM (all P > 0.05) (Table 2).

**Table 2:** Intraoperative data of the two groups.

	Before PSM (n = 119)				After PSM(n = 50)			
	TAR with FET (n = 88)	HAR (n = 31)	t/χ^2^	*P* value	TAR with FET (n = 25)	HAR (n = 25)	t/χ^2^	*P* value
AR surgery procedure, n (%)								
Bentall	12 (13.6)	4 (12.9)	0.011	0.918	3 (12.0)	3 (12.0)		1.000*
Wheat	5 (5.7)	3 (9.7)	0.584	0.445	2 (8.0)	3 (12.0)		1.000*
AAR	23 (26.1)	8 (25.8)	0.001	0.971	4 (16.0)	5 (20.0)	0.136	0.713
Sinus reconstruction + AAR	48 (54.6)	16 (51.6)	0.079	0.778	16 (64.0)	14 (56.0)	0.333	0.564
Operation time (h)	7.1 ± 1.2	6.7 ± 0.9	1.693	0.093	7.2 ± 1.1	6.6 ± 0.9	2.110	0.040
CBP time (min)	169.1 ± 33.5	147.5 ± 27.4	3.2271	< 0.001	177.4 ± 34.2	153.5 ± 30.2	2.6192	0.012
ACC time (min)	125.6 ± 19.5	94.5 ± 15.6	8.015	< 0.001	110.7 ± 17.3	95.6 ± 15.8	3.223	0.002
HCA time (min)	12.4 ± 1.9	0	36.237	< 0.001	11.7 ± 1.6	0	36.562	< 0.001
Transfusion								
Erythrocyte (U)	4.4 ± 0.9	3.1 ± 1.1	6.516	< 0.001	4.1 ± 1.0	3.3 ± 1.0	2.828	0.007
Plasma (ml)	585.4 ± 205.6	440.7 ± 196.1	3.409	< 0.001	605.2 ± 210.4	421.6 ± 187.2	3.260	< 0.001
Platelet (U)	0.64 ± 0.25	0.41 ± 0.21	4.582	< 0.001	0.62 ± 0.26	0.44 ± 0.22	2.642	< 0.011

*PSM* propensity score-matched, *TAR* total aortic arch replacement, *FET* frozen elephant trunk implantation, *HAR* hybrid II aortic arch replacement, *AR* aortic root, *AAR* ascending aorta replacement, *CPB* cardiopulmonary bypass, *ACC* aortic cross-clamp, *HCA* hypothermic circulatory arrest, *h* hour, *min* minute, *ml* milliliter, *U* unit.

*Indicates Fisher's exact test.

### Clinical outcome

During hospitalization, 13 patients (14.8%) died in the TAR with FET group, of which 5 patients died due to severe pulmonary infection, 3 patients due to multiple organ failure, 2 patients due to AKI, 1 patient due to cerebral infarction, 1 patient due to massive hemorrhage and 1 patient due to gastrointestinal bleeding. Only 2 patients (6.5%) died in the HAR group, of which 1 patient died due to AKI and the other patient died due to pulmonary infection. Before PSM, 24-h fluid drainage (645.5 ± 208.5 vs. 780.8 ± 210.4 ml, *P* = 0.002) was less in the HAR group than that in the TAR with FET group. Moreover, the incidence of acute liver injury (12.9 vs. 32.9%, *P* = 0.032), AKI (22.6 vs. 46.5%, *P* = 0.019), pulmonary infection (16.1 vs. 35.2%, *P* = 0.036) were lower and mechanical ventilation time (35.2 ± 11.4 vs. 47.4 ± 15.9 h, *P* < 0.001), ICU stay time (3.6 ± 2.1 vs. 6.2 ± 2.4 days, *P* < 0.001) and hospital stay time (13.2 ± 3.8 vs. 16.8 ± 4 0.6, *P* < 0.001) were shorter in the HAR group than that in the TAR with FET group. Analysis of the results after PSM were comparable to those before PSM, except for pulmonary infection (*P* = 0.185). The in-hospital mortality was lower in the HAR group than that in the TAR with FET group both before PSM (6.5 vs. 14.8%, *P* = 0.230) and after PSM (4.0 vs. 16.0%, *P* = 0.349), but without statistical significance (Table 3).

**Table 3:** Postoperative data of the two groups.

	Before PSM (n = 119)				After PSM (n = 50)			
	TAR with FET (n = 88)	HAR (n = 31)	t/χ^2^	*P* value	TAR with FET (n = 25)	HAR (n = 25)	t/χ^2^	*P* value
Fluid drainage in 24 h (ml)	780.8 ± 210.4	645.5 ± 208.5	3.086	0.002	770.9 ± 201.6	650.1 ± 210.4	2.073	0.044
LCOS (n, %)	6 (6.8)	1 (3.2)	0.534	0.465	2 (8.0)	1 (4.0)		1.000*
IABP (n, %)	2 (2.3)	0 (0.0)		0.490*	1 (4.0)	0 (0.0)		1.000*
ALI (n, %)	29 (32.9)	4 (12.9)	4.599	0.032	11 (44.0)	4 (16.0)	4.667	0.031
AKI (n, %)	41 (46.5)	7 (22.6)	5.491	0.019	12 (44.0)	5 (20.0)	4.367	0.037
CRRT (n, %)	10 (11.4)	1 (3.2)	1.810	0.179	3 (12.0)	1 (4.0)		0.609*
Pulmonary infection (n, %)	31 (35.2)	5 (16.1)	4.381	0.036	8 (32.0)	4 (16.0)	1.754	0.185
Tracheotomy (n, %)	9 (12.5)	2 (6.5)	0.390	0.533	3 (12.0)	2 (8.0)		1.000*
Thoracotomy hemostasis (n, %)	3 (6.9)	0 (0.0)		0.567*	1 (4.0)	0 (0.0)		1.000*
Gastrointestinal bleeding (n, %)	2 (2.3)	1 (3.2)		1.000*	0 (0.0)	1 (4.0)		1.000*
TND (n, %)	12 (13.6)	3 (9.7)	0.326	0.568	4 (16.0)	3 (12.0)		1.000*
PND (n, %)	1 (1.2)	0 (0.0)		1.000*	1 (4.0)	0 (0.0)		1.000*
Paraplegia (n, %)	2 (2.3)	1 (3.2)		1.000*	1 (4.0)	1 (4.0)		1.000*
Mechanical ventilation time (h)	47.4 ± 15.9	35.2 ± 11.4	3.926	< 0.001	45.5 ± 13.6	34.0 ± 10.9	3.299	0.002
ICU time (d)	6.2 ± 2.4	3.6 ± 2.1	5.350	< 0.001	5.9 ± 2.2	3.8 ± 2.2	3.375	0.002
Hospital stay time (d)	16.8 ± 4.6	13.2 ± 3.8	3.909	< 0.001	17.5 ± 5.3	13.0 ± 4.0	3.512	< 0.001
In-hospital mortality (n, %)	13 (14.8)	2 (6.5)	1.441	0.230	4 (16.0)	1 (4.0)		0.349*

*PSM* propensity score-matched, *TAR* total aortic arch replacement, *FET* frozen elephant trunk implantation, *HAR* hybrid II aortic arch replacement, *LCOS* low cardiac output syndrome, *IABP* intra-aortic ballon pump, *ALI* acute liver injury, *AKI* acute kidney injury, *CRRT* continuous renal replacement therapy, *TND* transient neurological dysfunction, *PND* permanent neurological dysfunction, *ICU* intensive care unit, *h* hour, *ml* milliliter, d day.

*Indicates Fisher’s exact test.

### Follow-up data

**Figure 4:**
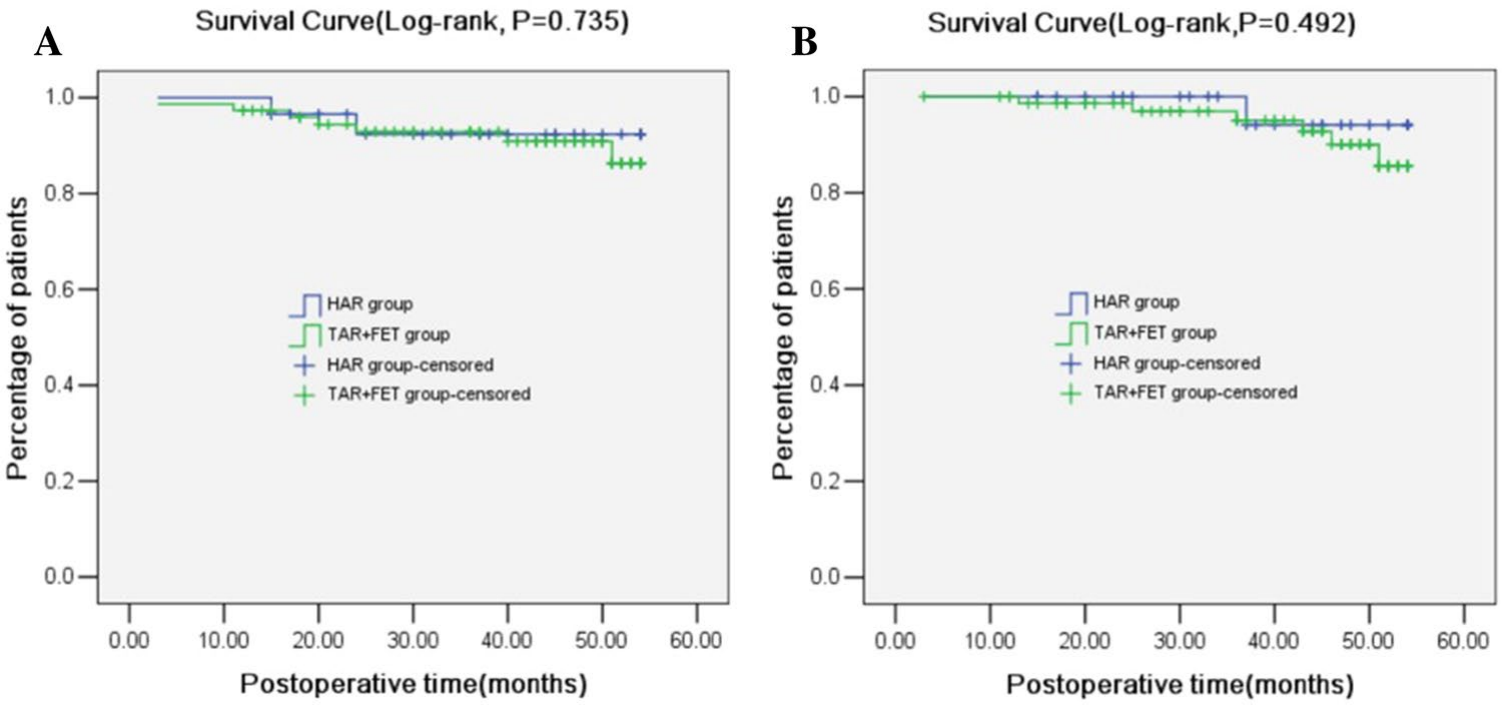
Kaplan–Meier analysis of follow-up mortality (**A**) and reoperation (**B**).

The mean follow-up period was 28 months (12–54 months). Three patients were lost to follow-up in the TAR with FET group while one patient was lost to follow-up in the HAR group. The follow-up rates were 96.0 and 96.6% in TAR with FET and HAR groups, respectively. During the follow-up period, 7 patients in the TAR with FET group, of which 2 patients died due to cerebrovascular accident, 1 due to gastrointestinal bleeding, 1 due to myocardial infarction and 3 due to an unknown reason. Notably, 2 patients died in the HAR group, of which 1 patient died due to cerebral hemorrhage and the other patient died due to lung cancer. The true lumen of the aortic arch (29.3 ± 1.5 vs. 27.1 ± 1.1 mm, *P* < 0.001) and middle descending thoracic aorta (23.7 ± 2.1 vs. 21.4 ± 1.9 mm, *P* < 0.001) were larger in the HAR group than in the TAR with FET group. False lumen thrombosis rates of the middle descending thoracic aorta (82.8 vs. 62.7%, *P* = 0.048) and renal artery level (37.9 vs. 28.7%, *P* = 0.039) were higher in the HAR group than in the TAR with FET group (Table 4). The follow-up mortality (3.4 vs. 8.0%, *P* = 0.692) and reoperation (6.9 vs. 9.3%, *P* = 0.406) were lower in the HAR group than in the TAR with FET group, but without statistical significance (Table 4). Kaplan–Meier analysis also showed that there were no significant differences between the two groups in follow-up mortality (*P* = 0.735, Fig. 4A) and reoperation (*P* = 0.492, Fig. 4B).

**Table 4:** Mid-term prognosis and follow-up results of the two groups.

	Before PSM (n = 119)				After PSM (n = 50)			
	TAR with FET (n = 88)	HAR (n = 31)	t/χ^2^	*P* value	TAR with FET (n = 25)	HAR (n = 25)	t/χ^2^	*P* value
Aortic diameter of true lumen								
Ascending aorta (mm)	26.3 ± 0.9	26.4 ± 1.0	-0.333	0.739	26.2 ± 0.9	26.3 ± 0.9	0.012	1.000
Aortic arch (mm)	27.1 ± 1.1	29.3 ± 1.5	-8.227	< 0.001	26.8 ± 1.2	29.1 ± 1.9	-5.117	< 0.001
Middle descending thoracic aorta (mm)	21.4 ± 1.9	23.7 ± 2.1	-5.374	< 0.001	21.6 ± 1.8	24.1 ± 1.9	-4.776	< 0.001
Renal artery level (mm)	19.2 ± 2.5	19.9 ± 2.7	-1.252	0.213	19.0 ± 2.3	19.9 ± 2.6	-1.296	0.201
Middle abdominal aorta level (mm)	18.1 ± 2.4	19.0 ± 2.5	-1.695	0.093	17.8 ± 2.3	19.1 ± 2.4	-1.955	0.056
False lumen thrombosis^#^								
Distal arch level (n, %)	71 (94.7)	28 (96.6)	0.162	0.687	23 (92.0)	25 (100.0)		1.000*
Middle descending thoracic aorta (n, %)	47 (62.7)	24 (82.8)	3.897	0.048	15 (60.0)	21 (84.0)	3.571	0.059
Renal artery level (n, %)	14 (18.7)	11 (37.9)	4.250	0.039	4 (16.0)	10 (40.0)	3.571	0.059
Middle abdominal aorta level (n, %)	9 (12.0)	6 (20.7)	1.279	0.258	2 (8.0)	5 (20.0)	1.495	0.221
Internal leakage (n, %)	6 (8.0)	2 (6.9)	0.036	0.850	2 (8.0)	1 (4.0)		1.000*
Follow-up mortality (n, %)	7 (9.3)	2 (6.9)	0.157	0.692	2 (8.0)	1 (4.0)		1.000*
Reoperation (n, %)	6 (8.0)	1 (3.4)	0.690	0.406	2 (8.0)	1 (4.0)		1.000*

*TAR* total aortic arch replacement, *FET* frozen elephant trunk implantation, *HAR* hybrid II aortic arch replacement.

^#^False lumen thrombosis is defined as thrombosis of > 80% or complete obliteration of the false lumen.

*Indicates Fisher’s exact test.

## Discussion

In this study, we compared the clinical outcomes and follow-up results of 88 elderly patients and 31 elderly patients with a-TAAD who underwent TAR with FET and two-stage HAR, respectively. PSM was employed to control for confounding factors. It was found that compared with the TAR with FET group, patients in the two-stage HAR group had less trauma, faster recovery, lower perioperative complications and mortality, better thoracic aortic remodeling and comparable medium-term follow-up mortality and readmission rate. These results indicate that two-stage HAR surgery is a safe and effective surgical procedure for elderly patients with a-TAAD.

A-TAAD is a serious disease with high mortality and is considered a surgical emergency. Studies have shown that the 48-h mortality rate of a-TAAD patients without surgery is as high as 50%^1,2^. The main causes of death are aortic rupture and failure of vital organs due to insufficient blood supply^11^. Elderly a-TAAD patients usually with special conditions^12^ such as preoperative complications, including chronic obstructive pulmonary disease, diabetes, cerebral infarction, organ dysfunction, etc., are more likely to incur organ injury after surgery. Moreover, a-TAAD patients with low autoimmune are at a high risk of postoperative infection, and those with poor coagulation function require a massive blood transfusion, resulting in an increased incidence of postoperative liver and kidney failure. The above factors contribute to the high postoperative mortality of elderly patients with a-TAAD. Several studies have shown that age is an independent risk factor for postoperative complications and mortality in a-TAAD patients^13–15^. Therefore, identifying a safe and effective surgical procedure is of great significance to reduce mortality and improve the prognosis of elderly patients with a-TAAD.

Clinical studies have confirmed that TAR with FET is the best surgical procedure for a-TAAD^4,5^. However, this surgical procedure is particularly complicated, needs to be completed under HCA and prolonged CPB and ACC times are inevitable, leading to increased risk of postoperative complications and mortality, especially in elderly patients or patients with malperfusion syndrome^6,16^. Prolonged CPB and ACC times are independent risk factors of adverse outcomes and mortality following cardiac surgery^17,18^, and HCA time is an independent risk factor of mortality in a-TAAD patients^19^. With the rapid development of interventional techniques in recent years, new concepts in the surgical treatment of a-TAAD have been proposed. In 2007, Szeto et al.^20^ first proposed hybrid total arch replacement and achieved success. Since then, HAR surgery has been widely used in aortic arch lesions. HAR combines open surgical debranching with TEVAR, preventing HCA and simplifying the operation, thereby reducing mortality^21^. Recently, the HAR technique has been widely employed in the treatment of a-TAAD, and achieved good short- and medium-term results^8–10^. A recent meta-analysis of 1305 patients from 11 studies compared the results of type II HAR and TAR with FET. They found that type II HAR was associated with reduced early mortality, stroke, spinal cord injury, renal impairment requiring dialysis and reoperation for bleeding and lung infection, verified the safety of type II HAR^22^. However, all studies used one-stage HAR, which is performed in hybrid operating rooms; however, most medical facilities in developing countries lack hybrid operating rooms. In such centers, conservative treatment may be used for elderly patients with more comorbidities combined with malperfusion syndrome or those who are unable to tolerate HCA, leading to poor prognosis. The current study explored the two-stage HAR procedure for high-risk elderly a-TAAD patients based on previous studies and achieved good results. CPB, ACC, HCA, mechanical ventilation, ICU stay and hospital stay times were shorter and the complications of acute liver injury, AKI and pulmonary infection were lower in the two-stage HAR group than that in the TAR with FET group, which were consistent with previous one-stage HAR studies^8–10^.

The HAR procedure has several advantages compared with the conventional TAR with FET. First, the HAR procedure prevents HCA and continuous cerebral perfusion and minimizes complications caused by organ ischemia, including brain, liver, kidney and other important organs. Second, distal anastomosis is located at the end of the ascending aorta, and the forward movement of anastomosis brings convenience to the operation, shortens CPB time and reduces anastomotic bleeding. Third, the HAR procedure prevents coagulation dysfunction caused by low temperature and reduced blood transfusion as well as the damage caused by blood transfusion. Finally, the descending aortic stent provides a wider coverage, better expansion of the true lumen, and good perfusion of important organs. Nonetheless, during the HAR procedure, strategies to reserve sufficient TEVAR anchoring area need to be developed.

In our study, adhesions behind the aortic arch were dissociated sufficiently and the occluding clamp was placed between the left common carotid artery and the left subclavian artery so that anastomosis was located at the end of the ascending aorta and even at the beginning of the aortic arch. For patients with a short ascending aorta and unable to provide more than 1.5 cm anchoring area after vascular prosthesis anastomosis, we propose anastomosis of the fourth branch of a vascular prosthesis to the left subclavian artery and ligation of the branch closest to the anastomosis to increase the anchoring area. A deeply located left subclavian artery that is difficult to anastomose should be ignored and left subclavian artery fenestration or chimney stenting should be performed during TEVAR. In our study, 2 patients underwent left subclavian artery fenestration, and 1 patient underwent left subclavian artery chimney stent implantation. We suggest early timing of interventional surgery after debranching, and TEVAR should be performed if two of the following three surgical indications are met: (1) hemodynamic stability, systolic blood pressure > 90 mmHg or mean arterial pressure > 60 mmHg; (2) recovery of consciousness, no cerebral complications; (3) drainage flow < 50 ml/h. In the current study, the time between the end of open surgery and the start of interventional surgery was 10.5 ± 3.4 h, and only 1 patient underwent TEVAR 7 days after open surgery due to consciousness disturbance. It is crucial to prevent the rupture of the aortic arch and descending aorta dissection during open surgery, and the two-stage HAR procedure is the preferred surgical intervention. In our perspective, gentle aortic arch occlusion is imperative during open surgery, and preventing excessive blood pressure fluctuations after the procedure plays a crucial role in reducing the risk of vascular rupture. The use of contrast media during TEAVR may theoretically cause or aggravate AKI. However, our study results revealed that the incidence of AKI (22.6 vs. 46.5%) and CRRT utilization (3.2 vs. 11.4) was lower in the two-stage HAR group than in the TAR with FET group, indicating that the use of contrast media during TEVAR was safe. Analysis of the medium-term follow-up results showed that the distal aortic remodeling was better, the true lumen of the distal descending aorta and abdominal aorta was significantly expanded while the rate of false lumen thrombosis was higher in the two-stage HAR group. This may be due to the stronger support force of the interventional stent and higher fitting degree of the vascular wall or the wider coverage of the descending aorta by the interventional stent.

## Limitation

This study has some limitations. Firstly, this is a single-center retrospective study, and recall bias is inevitable. Secondly, the sample size was small, which may affect the statistical results. Thirdly, the follow-up time was short, and therefore a long-term follow-up period should be considered in future studies. Finally, we used different types of stent grafts, which may influence the outcomes. Therefore, more rigorous multicenter prospective randomized controlled studies are needed to further corroborate our findings.

## Conclusion

Two-stage HAR is a safe and effective method for the treatment of elderly patients with a-TAAD. Compared with conventional TAR with FET, the two-stage HAR procedure has the advantages of less trauma, rapid recovery, lower incidence of complications and better aortic remodeling. It may be a good choice for elderly a-TAAD patients with comorbidities; however, future studies should be conducted with long-term follow-up periods.

## Data Availability

The datasets generated and analyzed during the current study are available from the corresponding author on reasonable request.
